# Perceived stigmatization and its impact on quality of life - results from a large register-based study including breast, colon, prostate and lung cancer patients

**DOI:** 10.1186/s12885-017-3742-2

**Published:** 2017-11-09

**Authors:** J. Ernst, A. Mehnert, A. Dietz, B. Hornemann, P. Esser

**Affiliations:** 1 Department of Medical Psychology and Medical Sociology, University Medical Center Leipzig, Philipp-Rosenthal-Str. 55, D – 04103 Leipzig, Germany; 2 Clinic of Otolaryngology, Head and Neck Surgery, University Medical Center Leipzig, Leipzig, Germany; 3 Tumor Center e.V., University Medical Center Leipzig, Leipzig, Germany; 4 University Cancer Center (UCC) Carl Gustav Carus, Technical University Dresden, Dresden, Germany

**Keywords:** Cancer, Stigmatization, Psycho-oncology, Quality of life, Survivorship

## Abstract

**Background:**

To date, research on stigmatization among cancer patients and related psychosocial consequences has been scarce and mostly based on small and highly selected samples. We investigated stigmatization and its impact on quality of life among a large sample including four major tumor entities.

**Methods:**

We assessed 858 patients with breast, colon, lung or prostate cancer from two cancer registries. Stigmatization and quality of life (QoL) was assessed with the Social Impact Scale (SIS-D) and the EORTC Quality of Life Questionnaire (European Organization for Research and Treatment of Cancer), respectively. Group effects were analyzed via analyses of variance, relationships were investigated via Pearson’s r and stepwise regression analyses.

**Results:**

The mean age was 60.7 years, 54% were male. Across cancer sites, the dimensions of stigmatization (isolation, social rejection, financial insecurity and internalized shame) were in the lower and middle range, with the highest values found for isolation. Stigmatization was lowest among prostate cancer patients. Stigmatization predicted all five areas of QoL among breast cancer patients (*p* < .05), but only affected emotional functioning (*p* < .01) among lung cancer patients.

**Conclusions:**

We found an inverse relationship between perceived cancer-related stigmatization and various dimensions of QoL, with variation between cancer sites. Breast cancer patients should be focused in individual therapies regarding the negative consequences accompanied by perceived stigmatization.

## Background

Health-related stigmatization is defined as a process by which a person is associated with negative properties due to his or her illness. As a result, the stigmatized person experiences devaluation by others and exclusion from social relationships [1]. Depending on the perspective, stigmatization can either mean stigmatizing attitudes and behaviors of a healthy person against ill persons or the perception and the consequences of stigmatization within the stigmatized person [2]. The negative consequences of perceived stigmatization can persist [3] and cause severe psychosomatic symptoms [4]. In many cases, the consequences of cancer-related stigmatization are even more distressing than the illness itself. In combination with social isolation and severe psychological and compliance problems, stigmatization finally results in a loss of quality of life (QoL) [5–8]. Therefore, investigation of stigmatization and its consequences among cancer patients is of great clinical importance.

So far, research on the extent of perceived stigmatization among cancer patients and its potential consequences has been scarce. Among specific and mixed cancer sites, perceived stigmatization ranges from 13% to 80% [9–12]. Among lung cancer patients, internalized feelings of guilt owing to preceding tobacco use contribute to heightened stigmatization [13], even though its extent does not differ from the level among head and neck cancer patients [5]. Among breast and prostate cancer patients, stigmatization is additionally influenced by the loss of the female or male identity or sexual functioning. However, similar to colon cancer, those body changes are not always visible and therefore do not lead to high distress first. In the long-term, however, they can cause severe distress, exerted via withdrawal from social relationships (e.g. from employment) and supporting tendencies for stigmatization [12].

Previous research is mostly based on lung cancer patients and showed significant associations (*r* > .5; *p* < .01) between health-related QoL and stigmatization [14–16]. In a recent study among lung cancer patients, Chambers, Baade et al. [17] found negative effects of stigmatization (internalized shame) on QoL (b = −.792, *p* < .05). The association between stigmatization (social rejection) and quality of life could also be shown among cancer patients with visible disfigurements (F = 2.55, *p* < .05) [18]. According to a review including 15 studies, the few studies on the relationship between stigmatization and QoL were mostly of low methodological quality [19], with most results based on small and highly selected samples.

Given the lack of research and methodological limitations of previous studies, further research of stigmatization using larger samples is needed. We present data from a large register-based study including 858 patients across four major tumor entities. We aimed to answer the following questions:

1. To what extent do cancer patients feel stigmatized? Do levels of stigmatization differ between dimensions on the Social Impact Scale (SIS-D) and groups by cancer site?
2. Are there significant associations between the level of stigmatization and QoL? Do these relationships differ between cancer sites?

## Methods

### Data collection

Data collection from two German cancer registries (cities of Leipzig and Dresden) was carried out between May and September 2016. Trained personnel in the cancer registries extracted patients according to the inclusion criteria, namely (i) age between 18 and 75 years, (ii) time of diagnoses not more than 30 months before and (iii) new diagnosis or relapse. The selection of patients was stratified by cancer site in order to create equally sized groups despite different incidence rates. In total, 1748 patients suffering from either breast, prostate, colon or lung cancer were contacted by mail and asked to fill out the pen and paper questionnaire. If patients did not respond, they were reminded twice and asked for either participation or reporting their reason for non-participation.

### Measures

#### Sociodemographic and medical data

Sociodemographic and medical data were assessed via self-report and included age, gender, marital and employment status, household income, time since diagnosis in years, UICC cancer stage, occurrence of metastases, type of cancer treatment and whether they were currently in treatment. The exact diagnoses according to the ICD-10 were transferred from the cancer registries.

#### Stigmatization

Perceived stigmatization was assessed with the validated German version of the Social Impact Scale (SIS-D) [20, 21], encompassing four dimensions named *isolation* (Cronbach’s α = .89; 9 Items, range 0-27), *social rejection* (Cronbach’s α = .81; 6 Items, range 0-18), *internalized shame* (Cronbach’s α = .81; 6 Items, range 0-18) and *financial insecurity* (Cronbach’s α = .81; 3 Items, range 0-9). Three of the four scales of the German version slightly differ from the original version [21]. Items are rated on a 4-point Likert scale ranging from *strong disagreement* to *strong agreement*. The aggregation of all items to a total value (range 0-72) is possible and showed excellent internal consistency with Cronbach’s α = .93 [21]. Examples of items are *I feel others avoid me because of my illness* (social rejection) or *I feel others think I am to blame for my illness* (internalized shame).

#### Quality of life

Health-related quality of life was assessed with the German version of the EORTC QLQ-C30, a multidimensional questionnaire of the European Organization for Research and Treatment of Cancer [22]. The instrument contains 30 items encompassing 5 functioning scales (cognitive, social, emotional, role, physical), 9 symptom scales (e.g. fatigue and pain) and single items (e.g. financial situation) as well as a global scale. The items are rated on 4-point Likert scale ranging from *not at all* to *very much* and on a 7-point Likert scale ranging from *very poor* to *excellent* (global scale). For our analyses, we focused on the five function scales, which were transformed to values ranging from 0 (worst functioning) and 100 (best functioning). Internal consistency (Cronbach’s α) of the five functioning scales ranges between .72 (cognitive functioning) and .90 (role functioning) [23]. Examples of items are *Do you have any trouble taking a long walk?* (physical functioning) or *Has your physical condition or medical treatment interfered with your family life?* (social functioning).

#### Depressive Symptomatology

The PHQ-9 is the depression module of the German version of the Patient Health Questionnaire (PHQ-D) [24], assessing depressive symptomatology with 9 items based on the DSM-IV criteria. The sum score can be used to determine severity of the depressive symptomatology. Internal consistency (Cronbach’s α) was .88 [24].

### Statistical analyses

Responders were compared to non-responders via chi-square tests (categorical variables) and t-tests for independent samples (variables with at least ordinal scale). Differences in stigmatization between cancer sites were investigated via ANOVA (1- and 2-factorial, including post-hoc-tests). Bivariate correlations between stigmatization and QoL were calculated via Pearson’s *r*. The effect of stigmatization on QoL when controlling for other variables (depressive symptomatology, time since diagnosis, gender and age) were tested with stepwise linear regression. The outcome variable in the main regression model was the stigmatization total score. Separate models were run for each dependent variable, i.e. each function scale. Alpha was two-sided and set at .05. Effect sizes were interpreted according to Cohen (d ≥ .2: small; d ≥ .5: medium; d > .8: large). All analyses were performed with SPSS Vs. 24. Fig. 2 was created with R Vs. 3.3.1.

## Results

### Sample characteristics

**Fig. 1:**
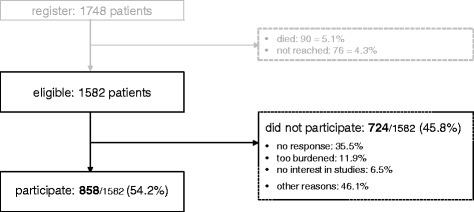
Flowchart of the sample

As illustrated in Fig. 1, 9.4% of the 1748 approached patients were deceased or could not be reached, leaving *N* = 1582 eligible patients. Of these patients, 858 participated at the study, leading to a response rate of 54%. Among the 724 non-participants, 65% reported their reasons for denial, the most frequent being “psychological burden” (11.9%) and “not interested” (6.5%). As presented in Table 1, responders and non-responders differed with respect to diagnosis: The frequency of breast and prostate cancer was higher among responders; the contrary result was found for colon and lung cancer patients (*p* = .023). Furthermore, responders had lower tumor stages (*p* = .033). Participants had a mean age of 60.7 years, 54.4% were male 49.7% were retired. 34.6% were diagnosed with breast, 31.2% with prostate, 19.6% with colon and 14.6% with lung cancer. The mean time since diagnosis was 1.9 years and 66% were currently treated.

**Table 1:** Sample characteristics and responder analyses

		Sample (*n* = 858)		non-responder ^a^ (*n* = 724)		t / chi^2^	*p*
Category		n	%	n	%		
Center	Leipzig	388	45.2%	308	42.5%	1.14	.286
	Dresden	470	54.8%	416	57.5%		
Age (years)	mean (sd, range)	60.7 (9.3, 23-73)		60.4 (9.6, 26-74)		.26	.607
Sex	male	467	54.4%	402	55.5%	.19	.685
	female	391	45.6%	322	44.5%		
Marital status	single	90	10.5%	–	–	–	–
	married	626	73.0%				
	divorced	86	10.0%				
	widowed	54	6.3%				
	missing data	2	.2%				
Employment	employed	360	42.0%	–	–	–	–
	retired	426	49.7%				
	unemployed	20	2.3%				
	other	24	2.8%				
	missing data	28	3.3%				
Household income (€/month)	< 2000	353	41.1%	–	–	–	–
	2000-3000	266	31.0%				
	> 3000	205	23.9%				
	missing data	34	4.0%				
Cancer site (ICD-10)	breast (C50)	297	34.6%	220	30.4%	9.52	.023
	colon (C26)	168	19.6%	160	22.1%		
	lung (C34)	125	14.6%	139	19.2%		
	prostate (C61)	268	31.2%	205	28.3%		
Time since diagnosis (years)	mean (sd, range)	1.9 (1.9, 0-28)		1.7 (.75, 0-3)		3.13	.077
	missing data	38	4.4%	308	57.5%		
UICC ^b^	I	162	18.9%	108	14.9% ^b^	8.73	.033
	II	71	8.3%	35	4.8%		
	III	83	9.7%	38	5.2%		
	IV	47	5.5%	38	5.2%		
	missing data	495	57.7%	89	29.9%		
Metastases	no	640	74.6%	–	–	–	–
	yes	175	20.4%				
	missing data	43	5.0%				
Currently in treatment	no	242	28.2%	–	–	–	–
	yes	565	65.9%				
	missing data	51	5.9%				
Type of treatment (yes) ^c^	chemotherapy	367	42.8%	–	–	–	–
	radiotherapy	522	60.8%				
	operation	607	70.7%				

^a^ Owing to data protection, medical information for non-responders is available only for a couple of variables

^b^ Data available only for patients from the cancer registry of Leipzig

^c^ Combinations possible

### Extent of perceived stigmatization for each subscale and cancer site

**Fig. 2:**
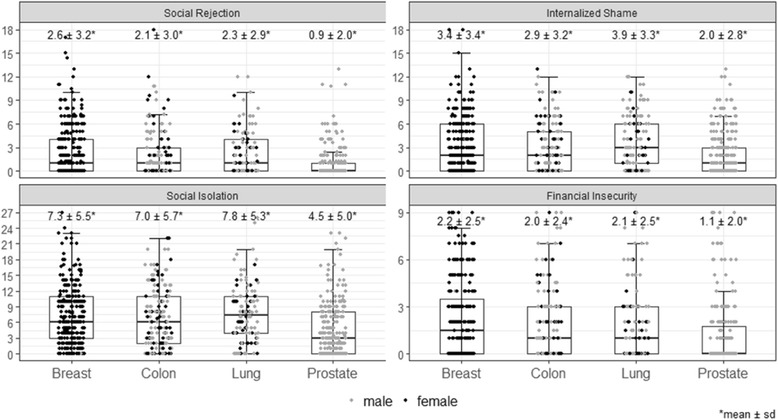
Perceived stigmatization for each dimension and cancer site. Note: Depending on the respective number of items, scales are ranging between 9 and 27

As presented in Fig. 2, the mean level of stigmatization in each dimension was in the lower or middle range. Mean scores were lowest for social rejection and internalized shame and higher for isolation and financial insecurity. Analyses of variance revealed that prostate cancer patients showed significantly lower levels in all dimensions compared to the other groups, namely social rejection (*p* < .001; d = .48 - .63), isolation (*p* < .001; d = .31 - .65), financial insecurity (*p* < .002; d < .5) and internalized shame (*p* < .05; d = .31-.63). Group effects were largest between prostate and lung cancer patients.

Since cancer site was highly confounded with gender (prostate cancer: all male; breast cancer: almost all female), we investigated whether differences between diagnosis represent gender effects. Therefore, we conducted a 2-factorial analysis of variance for the mixed gender groups (colon and lung cancer). These analyses showed only one significant effect, namely higher values for males in financial insecurity (*p* = .018). No further differences or interactions between gender and cancer group in the dimensions were observed. Therefore, we assumed that the shown differences between cancer groups are not caused or biased by gender effects.

### Relationships between stigmatization and quality of life

As presented in Table 2, all bivariate correlations between the dimensions of stigmatization and the different function scales of QoL were ≥ .31, with all *p* < .001.

**Table 2:** Bivariate associations between stigmatization and quality of life (Pearsons r)

		Quality of life*				
		Physical	Role	Emotional	Cognitive	Social
Stigmatization	Rejection	−.31	−.33	−.47	−.35	−.43
	Finances	−.33	−.39	−.47	−.37	−.41
	Shame	−.32	−.35	−.47	−.38	−.43
	Isolation	−.51	−.54	−.61	−.50	−.61
	Total value	−.45	−.51	−.61	−.48	−.57

* all r significant with *p* < .001

When controlling for depressive symptomatology, age, gender (only for colon and lung cancer) and time since diagnosis, stigmatization showed a negative impact on all areas of QoL among breast cancer patients (all *p* < .001 except for cognitive functioning). Among lung cancer patients, stigmatization only affected emotional functioning (*p* < .01). In colon cancer patients, significant effects were found for role, social (*p* < .001) and emotional (*p* < .01) functioning. Among prostate cancer patients, stigmatization significantly influenced physical, role (*p* < .01) and social (*p* < .001) functioning.

Significant effects on QoL were also found for depressive symptomatology, which were higher than for stigmatization (standardized Beta; all *p* < .001). Further significant effects were found for age in breast cancer patients (negative impact on physical functioning with *p* < .001and positive impact on cognitive functioning with *p* < .01) and time since diagnosis in prostate cancer patients (positive impact on social functioning with *p* < .01). Gender was excluded in each of the models owing to non-significant effects in the stepwise processes. Explained variance in each model was acceptable ranging from 32% to 70%, being highest for emotional (60.3% to 70%) and lowest for physical (32% to 47.9%) functioning. Table 3 shows all models.

**Table 3:** Multivariate models with stigmatization as a significant predictor for QoL

	Predictors	B	SD B	Stand. Beta	*p*
Physical functioning					
Breast (*n* = 275)	Depression	−1.948	.247	−.451	<.001
	Age	−.635	.107	−.296	<.001
	Stigmatization	−.395	.101	−.234	<.001
Prostate (*n* = 240)	Depression	−1.659	.280	−.412	<.001
	Stigmatization	−.356	.117	−.211	<.01
Role functioning					
Breast (*n* = 273)	Depression	−2.822	.297	−.514	<.001
	Stigmatization	−.507	.116	−.236	<.001
Colon (*n* = 153)	Depression	−2.209	.531	−.357	<.001
	Stigmatization	−.775	.205	−.323	<.001
Prostate (n = 240)	Depression	−2.862	.344	−.525	<.001
	Stigmatization	−.448	.144	−.196	<.01
Emotional functioning					
Breast (*n* = 277)	Depression	−3.324	.254	−.599	<.001
	Stigmatization	−.573	.099	−.265	<.001
Colon (n = 153)	Depression	−3.418	.347	−.650	<.001
	Stigmatization	−.409	.134	−.201	<.01
Lung (*n* = 102)	Depression	−3.443	.346	−.671	<.001
	Stigmatization	−.605	.169	−.242	<.01
Cognitive functioning					
Breast (n = 277)	Depression	−3.524	.297	−.604	<.001
	Age	.343	.128	.119	<.01
	Stigmatization	−.304	.121	−.134	<.05
Social functioning					
Breast (*n* = 276)	Depression	−2.109	.374	−.335	<.001
	Stigmatization	−.787	.145	−.322	<.001
Colon (n = 153)	Depression	−2.918	.536	−.426	<.001
	Stigmatization	−.897	.207	−.338	<.001
Prostate (n = 240)	Depression	−1.972	.364	−.320	<.001
	Stigmatization	−1.169	.152	−.453	<.001
	Time since diagnosis	2.386	.806	.134	<.01

## Discussion

The present study investigated the relationship between perceived stigmatization and health-related QoL among 858 cancer patients across four major tumor entities. We found that the level of perceived stigmatization was in the lower and middle range for all dimensions and slightly varied between cancer sites. Associations between stigmatization and different domains of QoL were shown for each cancer site, but were most extensive among breast cancer patients.

Our findings regarding the relatively low values of stigmatization correspond to the German validation study of the SIS [21], which is even below our results. These differences can partially be explained by the relatively small sample (*n* = 139) and the inclusion of several other cancer sites in their study, e.g. hematological malignancies or genital tumors. However, to estimate whether the levels of stigmatization are problematic, representative studies among the general population would be needed. Furthermore, given the far reaching adverse effects of stigmatization, including the timing of health seeking behavior, even low levels are highly relevant and should be taken seriously.

Our study further shows that patients scored highest in isolation. Isolation scores were particularly high in lung cancer patients. Lung cancer patients also reported the highest levels of internalized shame. This differences between lung cancer compared to other cancer sites is consistent with a study by Else-Quest, LoConte et al. [25] among lung, prostate and breast cancer patients, showing higher stigmatization among lung cancer patients. These elevated levels could be caused by feelings of guilt for having smoked [13, 26]. However, it has to be noted that we could only show group differences between lung and prostate cancer patients. Higher stigmatization in men, which were shown in previous studies [5, 13] could only be replicated for financial insecurity. Given that previous studies are mostly based on small and highly selected samples, our findings provide robust evidence that lung cancer patients are experiencing higher levels of stigmatization.

We further found significant negative associations between stigmatization and all subscales of QoL, which could be replicated across all dimensions of stigmatization and areas of QoL. This is in line with previous studies, reporting relationships of the same magnitude [14, 15, 17]. Most importantly, the effect of stigmatization on QoL was also found after controlling for covariates such as depressive symptomatology. This shows the relative importance of stigmatization in the wide range of possible predictors of QoL. Furthermore, the effect of stigmatization was highest in predicting the emotional, social and role area of QoL. This suggests that stigmatization is a phenomenon mostly associated with the behavioral and interactional dimensions of QoL.

However, we also observed that the effect of stigmatization on QoL depends on the cancer site. For example, stigmatization predicted all areas of QoL among breast cancer, but only emotional functioning among lung cancer patients. This could indicate that breast cancer patients might suffer from stigmatization more severely than other patient groups. Lung cancer patients, however, could be more used to stigmatization and therefore desensitized (e.g. by having a smoking history). However, aside from such interpretations, it is also possible that the significance and magnitude of the effects is directly linked to different test powers due to the different sample sizes (Breast: *n* = 297; Lung: *n* = 125).

Depressive symptomatology significantly predicted QoL in all models, which is consistent with previous studies, particularly studies with lung cancer patients [5, 7]. The interrelationship between stigmatization, depressive symptomatology and QoL indicates that the effect of stigmatization on QoL could be mediated by depressive symptomatology [17]. Mediator analyses are highly warranted to validate such hypotheses. Such studies should also control for other factors predicting psychosocial distress such as tumor stage and metastases, which were associated with distress in our study.

A major limitation of the study is the cross-sectional design. To show cause and effect relationships, longitudinal studies are highly warranted. Furthermore, our four homogenous patient groups heighten internal validity but at the same time reduce the generalizability of our results for other cancer entities. However, our large sample including two register-based sources provides robust results in a research field which is rarely investigated. Data on psychosocial symptom burden before the cancer diagnosis was not available, which did not allow for non-responder analyses in terms of such distress.

## Conclusions

Our results show that illness-related stigmatization among cancer patients is associated with considerable impairments in a wide range of areas of quality of life. Therefore, reducing stigmatization in cancer patients may lead to lower risks of developing longstanding psychological and psychosocial problems. Given our results, lung cancer patients should be focused in campaigns for destigmatization, whereas breast cancer patients should be focused in individual therapies aiming at negative consequences related to cancer-related stigmatization.

## Abbreviations

PHQ-9: Patient health questionnaire
QoL: Quality of life
SIS: Social impact scale
